# A Pure Electric Driverless Crawler Construction Machinery Walking Method Based on the Fusion SLAM and Improved Pure Pursuit Algorithms

**DOI:** 10.3390/s23187784

**Published:** 2023-09-10

**Authors:** Jiangdong Wu, Haoling Ren, Tianliang Lin, Yu Yao, Zhen Fang, Chang Liu

**Affiliations:** 1 College of Mechanical Engineering and Automation, Huaqiao University, Xiamen 361021, China; 1711121029@stu.hqu.edu.cn (J.W.); ltl@hqu.edu.cn (T.L.); 20014080010@stu.hqu.edu.cn (Z.F.); 22014080052@stu.hqu.edu.cn (C.L.); 2 Fujian Key Laboratory of Green Intelligent Drive and Transmission for Mobile Machinery, Xiamen 361021, China; 3 Mechatronic Engineering with the School of Beihang University, Beijing 102206, China; 19013080044@stu.hqu.edu.cn

**Keywords:** crawler construction machinery, unmanned driving, fusion SLAM, improved pure pursuit algorithm

## Abstract

Driverless technology refers to the technology that vehicles use to drive independently with the help of driverless system under the condition of unmanned intervention. The working environment of construction machinery is bad, and the working conditions are complex. The use of driverless technology can greatly reduce the risk of driver operation, reduce labor costs and improve economic benefits.Aiming at the problem of the GPS positioning signal in the working environment of construction machinery being weak and not able to achieve accurate positioning, this paper uses the fusion SLAM algorithm based on improved NDT to realize the real-time positioning of the whole vehicle through reconstruction of the scene. Considering that the motion characteristics of crawler construction machinery are different from those of ordinary passenger cars, this paper improves the existing pure pursuit algorithm. Simulations and real vehicle tests show that the algorithm combined with the fusion SLAM algorithm can realize the motion control of driverless crawler construction machinery well, complete the tracking of the set trajectory perfectly and have high robustness. Considering that there is no mature walking method of driverless crawler construction machinery for reference, the research of this paper will lay a foundation for the development of driverless crawler construction machinery.

## 1. Introduction

With the aggravation of the “greenhouse effect” and the outbreak of the global energy crisis, traditional fuel construction machinery has been unable to meet the development needs of the times, and energy conservation and emission reduction of construction machinery has become the consensus in the industry [1]. Compared with traditional construction machinery, pure electric construction machinery has the advantages of high efficiency, low noise and no pollution [2]. At present, it has become one of the mainstream directions in the development of construction machinery industry. Compared with fuel construction machinery, it is easier to access the electrical components and driverless systems of pure electric construction machinery, achieving more accurate control.

Intelligentization of construction machinery includes intelligentization of products and construction, intelligentization of manufacturing, intelligentization of service and intelligentization of management, while driverless construction machinery belongs to the intelligentization module of products and construction [3]. Driverless construction machinery refers to construction machinery that can complete work and work path planning independently without human intervention [4]. At present, driverless cars are developing rapidly. Tesla, Waymo, Uber, Toyota, Baidu Apollo, Huawei Arcfox, Pony AI, Xpeng Inc and other well-known car companies have developed their own driverless cars and completed road tests of urbanization structures in a variety of bad weather [5]. Compared with the rapid development of driverless vehicles, the research on driverless construction machinery in the industry started late. Construction machinery is different from ordinary cars. It runs on unstructured roads, and thus it is difficult to extract road features. At the same time, the control system of construction machinery is also quite different from ordinary cars. Based on the above factors, the driverless system of an automobile is not suitable for construction machinery. Therefore, it is highly important for the intelligent process of construction machinery to develop a set of driverless systems suitable for construction machinery. Caterpillar, Komatsu and other world-famous construction machinery companies have been developing driverless construction machinery. The driverless mining dump truck developed by Komatsu has even canceled the cab, and it has been put into use in mining areas in Australia and Chile. Relatively speaking, the research on driverless construction machinery in China is still in its infancy, and most of it is based on remote control. There are few self-driving construction machinery products based on multi-sensor and deep learning methods [6].

Intelligent construction machinery generally uses GPS to achieve vehicle positioning, but GPS satellite positioning signals are easily obstructed and shielded by obstacles. In the working conditions of tunnels and buildings, GPS cannot provide accurate positioning information for the whole vehicle. Aiming at the problem of weak GPS positioning signals in some working environments of construction machinery [7], a fusion SLAM algorithm [8] based on improved NDT [9] is proposed in this paper. The algorithm collects environmental information through LiDAR and constructs a point cloud map. The real-time positioning of the whole vehicle is realized by matching the real-time perceived point cloud information with the constructed point cloud map. At the same time, the algorithm combines an IMU and odometer to make up for the slow mapping speed and cumulative error of single LiDAR SLAM. This set of positioning algorithms relies on LiDAR to construct geometric feature constraints, supplemented by an IMU and odometer to provide initial pose estimation values, and it can effectively solve the problem of vehicle positioning for construction machinery under conditions where satellite positioning signals are obstructed, such as in tunnels and buildings. Considering that crawler construction machinery belongs to the differential motion machinery, and the existing pure pursuit algorithm is only applicable to motion machinery based on Ackerman steering, this paper improves the existing pure pursuit algorithm and modifies its motion model to make it suitable for motion control of crawler construction machinery. Based on ROS, this paper builds a driverless simulation platform for crawler construction machinery and uses rviz to visualize the simulation effect. In order to verify the effect of the algorithm on real crawler construction machinery, a pure electric crawler excavator driverless platform is built, and the established path tracking experiment based on the fusion SLAM and improved pure pursuit algorithms is carried out.

## 2. Fusion SLAM System Based on Improved NDT

The driverless system includes five modules: perception, localization, planning, decision making and motion control [10]. The driverless system receives the environmental perception and positioning information through the multi-sensor platform and uses the computing platform to analyze the received perception and positioning information and perform corresponding planning and decision making. After the planning and decision making are completed, the computing platform sends control signals to the vehicle control system to complete the motion control of the vehicle. To sum up, in the driverless system, positioning information is very important, being the basis of planning, decision making and motion control.

At present, the commonly used multi-sensor fusion methods include the weighted average method, Kalman filtering method [11], multi-Bayesian estimation method and D-S evidence inference method. Gao et al. [12] proposed a distributed state fusion method based on sparse grid orthogonal filtering and established a matrix-weighted distributed state fusion method in the sense of the mean squared error, which improves the fusion performance of multi-sensor nonlinear systems. Gao et al. [13] proposed a new multi-sensor data fusion method for INS-, GPS- and SAR-integrated navigation systems. This method combines local scattered fusion with global optimal fusion, improving the accuracy and reliability of the integrated navigation system. Lai et al. [14] proposed a multi-sensor tight fusion method that combines inertial navigation systems (INSs), odometers, barometric altimeters and GNSS technology. In the absence of satellite signals, the accuracy of this method can be close to that of outdoor environments. Gao et al. [15] proposed a new random weighted estimation method for optimal fusion of multidimensional position data. This method can effectively fuse multi-sensor dimensional position data, and the fusion accuracy is much higher than the Kalman fusion method. Zong et al. [16] proposed a randomly weighted CKF (RWCKF) to handle the limitations of the CKF, overcoming the CKF problem and improving the accuracy of system state estimation. Yu et al. [17] proposed a distributed finite-time optimal fusion filtering method based on dynamic communication weights for distributed state estimation in sensor networks which achieves fusion error convergence and optimal estimation with minimal variance within a limited number of iterations.

In summary, this article combines RTK and INS data based on extended Kalman filtering to output the initial odometer information. Then, the initial odometer information is weighted and fused with the pose information obtained from IMU integration, and the initial pose information used for point cloud registration is finally output to improve the speed and accuracy of point cloud registration.

The SLAM system is mostly used for unmanned vehicle positioning in passenger cars. Up to now, it has formed five positioning systems: visual SLAM, laser SLAM, visual inertial navigation SLAM, laser inertial navigation SLAM and visual laser inertial navigation SLAM. Koestler et al. [18] proposed a dense map construction method based on monocular cameras (TANDEM). This method is based on a new front-end odometer estimation and a BA optimization algorithm, which can complete dense map construction with rich texture information using only low-cost monocular cameras. However, the amount of front-end matching data is too large to be deployed on real vehicles. Chen et al. [19] proposed a LiDAR positioning method based on a Monte Carlo and particle filter (Range MCL), which can accurately estimate the attitude of mobile robots or autonomous vehicles and achieve the global positioning of the whole vehicle, but there is no loop closure mechanism and it cannot control the global error well. Xu et al. [20] proposed a fast, robust and universal laser inertial odometer framework (FAST-LIO2), which uses an extended Kalman filtering algorithm based on multi-sensor tightly coupled iteration. It uses a direct registration method between the original point cloud of the current frame and the local map to solve the pose of the current frame, avoiding the computational waste caused by feature extraction and preventing the loss of detailed features of the original point cloud, effectively improving the real-time accuracy of point cloud registration. However, without the ability to resist degradation, it is prone to drift in localization and mapping in scenes such as tunnels, bridges and corridors.

In this paper, the fusion SLAM method based on improved NDT is used to obtain the real-time positioning information of the whole vehicle, which can still provide accurate positioning information of the whole vehicle for the computing platform without a GPS positioning signal [21]. Its advantage lies in the use of LiDAR to directly construct geometric feature constraints for point cloud registration without the need for feature extraction, greatly improving algorithm efficiency and ensuring real-time vehicle positioning. At the same time, the IMU and odometer are used to provide the initial values for point cloud registration, greatly accelerating the speed and accuracy of solving the vehicle’s position. It can still achieve high-precision localization and mapping without loop closure and has a certain degree of anti-degradation ability.

### 2.1. Point Cloud Registration Algorithm Based on NDT

#### 2.1.1. Point Cloud Registration

In the process of building a LiDAR point cloud map, because the scanning distance of LiDAR is limited, the point cloud data obtained by one scan cannot contain all the environmental information, and with the increase in distance, the obtained point cloud information will become more and more sparse. Therefore, in the process of mapping, the whole vehicle equipped with LiDAR needs to move continuously in the environment required for mapping, use LiDAR to collect enough environmental information, splice the collected point cloud data frame by frame and finally make a point cloud map. Point cloud registration is the process of converting the scanned point clouds of different frames in the same environment to the same coordinate system through a coordinate transformation matrix. The key of point cloud registration is to find the optimal coordinate transformation matrix between different frame point clouds. At present, the commonly used methods are the iterative nearest point (ICP) method and normal distribution transformation (NDT) method. The coordinate transformation matrix solved by the iterative nearest point method has high accuracy, but the disadvantage is that the amount of calculation is too large to meet the real-time requirements. The normal distribution transformation is based on the statistical principle, dividing the point cloud of the previous frame through the grid, assuming that the point cloud in the grid obeys the normal distribution, calculating the probability value of the converted point cloud in the grid of the point cloud division of the previous frame, multiplying all the probability values as the target likelihood function and using the Newton iteration method to continuously optimize the coordinate transformation matrix until the target likelihood function reaches the maximum value, At this time, the coordinate transformation matrix is the optimal coordinate transformation matrix [22]. However, the traditional NDT algorithm uses the Newton method to iterate, and the Hessian matrix of the objective function in its iterative equation is usually difficult to solve, which seriously affects the real-time performance of localization and mapping. Therefore, the traditional NDT algorithm is improved by the Levenberg–Marquardt iterative method [23]. The advantage of this method is that the Jacobian matrix of the objective function is used to approximate the Hessian matrix, which greatly improves the computational efficiency. At the same time, the added diagonal operator solves the singularity problem of the approximate Hessian matrix.

#### 2.1.2. NDT Algorithm

NDT is a model based on a statistical principle. If a group of random vectors obeys a normal distribution, then its probability density function is [24]

(1) $$p\left(x\right)=\frac{1}{{\left(2\pi \right)}^{\frac{D}{2}}\sqrt{\left|\sum \right|}}\exp\left(\frac{{\left(x-\vec{q}\right)}^{T}\sum^{-1}\left(x-\vec{q}\right)}{2\sigma^{2}}\right)$$

where the parameter *D* represents dimension, $\vec{q}$ represents the mean vector and ∑ represents the covariance matrix of a random vector. NDT uses probability to reflect the distribution of point clouds, which greatly reduces the registration time.

#### 2.1.3. NDT Algorithm Flow

**Figure 1:**
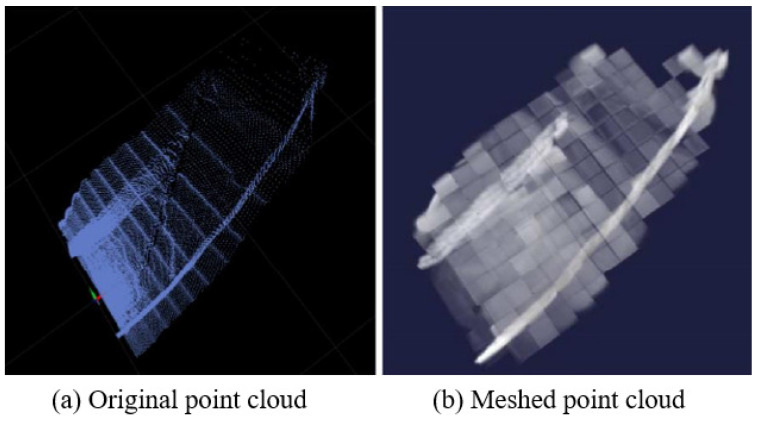
3D original point cloud and its meshed effect.

Point cloud registration generally refers to the splicing of point clouds of the upper and lower frames. The point cloud of the previous frame is generally the target point cloud, and the point cloud of the next frame is the source point cloud. During registration, the target point cloud is fixed, and the source point cloud is transferred to the coordinate system of the target point cloud through the coordinate transformation matrix. In this paper, the initial value of the coordinate transformation matrix is jointly provided by the Odom (odometer) and IMU, which can improve the speed and accuracy of mapping. The source point cloud is compared with the target point cloud after transformation of the initial coordinate transformation matrix, and the total score is calculated. The total score is finally maximized through the Levenberg–Marquardt iteration method. At this time, the corresponding coordinate transformation matrix is the optimal coordinate transformation matrix. This paper compares the target point cloud with the source point cloud after coordinate conversion based on the improved NDT algorithm. The first step of the NDT algorithm is to mesh the target point cloud (i.e., divide the target point cloud with a grid of the same size), as shown in Figure 1 [25].

The second step of the NDT algorithm is to load the target point cloud data. We put the target point cloud into the grid divided in the previous step and calculate the mean value of the point cloud data in each grid with Equation (2):

(2) $$\vec{q}=\frac{1}{m}\sum_{k=1}^{m}\vec{x_{k}}$$

where ${\vec{x}}_{k\;=\;1,2,3,...,m}$ represents the coordinates of all points of the point cloud in a single grid.

We use Equation (3) to solve the covariance of the point cloud data in each grid and finally use Equation (4) to solve the probability density function of the point cloud distribution in each grid:

(3) $$\sum =\frac{1}{m}\sum_{k=1}^{m}\left(\vec{x_{k}}-\vec{q}\right){\left(\vec{x_{k}}-\vec{q}\right)}^{T}$$

(4) $$p\left(\vec{x}\right)=\frac{1}{2^{\frac{3}{2}}\sqrt{\left|\sum \right|}}\exp\left(-\frac{{\left(\vec{x}-\vec{q}\right)}^{T}\sum^{-1}\left(\vec{x}-\vec{q}\right)}{2}\right)$$

The third step of the NDT algorithm is to solve the coordinate transformation matrix of the source point cloud relative to the target point cloud. The coordinate transformation matrix is generally composed of a translation matrix and a rotation matrix. The translation matrix describes the translation of the origin in the source point cloud coordinate system relative to the origin in the target point cloud coordinate system in the *x*, *y* and *z* directions. The rotation matrix is the rotation angle of the origin in the source point cloud coordinate system relative to the origin in the target point cloud coordinate system around the *x*, *y* and *z* axes, for which the industry usually uses roll, pitch and yaw to express them, respectively. Here, γ, β and α are used to correspond to the roll, pitch and yaw, respectively. Therefore, the expression of a rotation matrix $R_{3\times 3}$ is

(5) $$R_{3\times 3}=\left[\delta \;\varepsilon \;\eta \right]$$

The expressions of δ, ε and η are as follows:

(6) $$\delta =\left[\begin{array}{l} \cos\beta \cos\alpha \\ \sin\alpha \cos\beta \\ -\sin\beta \end{array}\right]$$

(7) $$\varepsilon =\left[\begin{array}{c} \cos\alpha \sin\beta \sin\gamma -\sin\alpha \cos\gamma \\ \cos\alpha \cos\gamma +\sin\alpha \sin\beta \sin\gamma \\ \cos\beta \sin\gamma \end{array}\right]$$

(8) $$\eta =\left[\begin{array}{c} \cos\alpha \sin\beta \cos\gamma +\sin\alpha \sin\gamma \\ \sin\alpha \sin\beta \cos\gamma -\cos\alpha \sin\gamma \\ \cos\gamma \cos\beta \end{array}\right]$$

The translation vector *t* is shown in Equation (9):

(9) $$t={\left[t_{x},t_{y},t_{z}\right]}^{T}$$

Assuming that the positions of a laser point in the target point cloud coordinate system and the source point cloud coordinate system are as illustrated in Equation (10) and Equation (11), respectively, the coordinate transformation relationship in Equation (13) between the same laser point in the target point cloud coordinate system and the source point cloud coordinate system can be obtained from Equation (12), where *T* is the coordinate transformation matrix of the source point cloud coordinate system relative to the target point cloud coordinate system:

(10) $$X^{\prime }=\left[\begin{array}{c} x_{i}^{{}^{\prime }}\\ y_{i}^{{}^{\prime }}\\ z_{i}^{{}^{\prime }} \end{array}\right]$$

(11) $$X=\left[\begin{array}{c} x_{i}\\ y_{i}\\ z_{i} \end{array}\right]$$

(12) $$X^{\prime }=R_{3\times 3}X+t_{3\times 1}=TX$$

(13) $$X^{\prime }=\left[\begin{array}{c} x_{i}^{{}^{\prime }}\\ y_{i}^{{}^{\prime }}\\ z_{i}^{{}^{\prime }} \end{array}\right]=\left[\delta \;\varepsilon \;\eta \right]\left[\begin{array}{c} x_{i}\\ y_{i}\\ z_{i} \end{array}\right]+\left[\begin{array}{c} t_{x}\\ t_{y}\\ t_{z} \end{array}\right]$$

In the operation process, it is necessary to give a good initial value to the above operation formula (i.e., the initial coordinate transformation matrix close to the optimal coordinate transformation matrix). This can accelerate the speed of NDT inter-frame matching, reduce the number of Levenberg–Marquardt iterations, reduce the computing pressure of the computing platform and achieve faster mapping. In the actual mapping process, the position and attitude information of the real vehicle are generally collected through the GNSS, IMU, Odom and other sensors, and the coordinate transformation matrix relative to the target point cloud coordinate system is obtained through coordinate transformation via the initial coordinate transformation matrix. Using the IMU or Odom alone to provide initial values will produce cumulative errors, which will affect the final localization and mapping. Therefore, this paper uses an IMU and Odom to provide the initial coordinate transformation matrix for inter-frame matching, in which the IMU provides attitude information and the Odom provides position information.

The fourth step of the NDT algorithm is to solve the probability of the source point cloud in the target point cloud grid after coordinate transformation. After the initial coordinate transformation matrix transformation, the source point cloud rotates and shifts to the target point cloud coordinate system, and it is divided in each grid by the target point cloud. $X^{\prime }$ represents the coordinates of a laser point in the source point cloud converted to the target point cloud coordinate system through the coordinate transformation matrix. Using the point cloud distribution probability density function of the grid where the laser point $X^{\prime }$ is located, the probability value that the coordinate of the laser point in the source point cloud is $X^{\prime }$ after coordinate transformation is solved. Similarly, the probability values of other laser points are solved.

The fifth step of the NDT algorithm is to solve the maximum objective likelihood function. The maximum objective likelihood function is obtained by multiplying the probability values of each laser point solved in the fourth step. When the maximum objective likelihood function reaches the maximum value (i.e., when the probability value of each laser point reaches the maximum), the coordinate transformation matrix at this time is considered to be the optimal solution. The expression of the maximum objective likelihood function is

(14) $$\psi =\prod_{k=1}^{n}p\left(X^{\prime }\right)$$

Since the maximum objective likelihood function is a multiplicative function, it is not easy to derive, and the subsequent optimization needs to derive the maximum objective likelihood function. Therefore, we use logarithms on both sides of the maximum objective likelihood function to simplify the function:

(15) $$-\log\psi =-\sum_{k=1}^{n}\log\left(p\left(X^{\prime }\right)\right)$$

The last step of the NDT algorithm is to solve the optimal coordinate transformation matrix with the Newton iteration method. In this paper, the Levenberg–Marquardt iteration method is used to replace the Newton iteration method, which not only improves the real-time performance of the localization and mapping system but also enhances the robustness. The core of solving Equation (15) with the Lievenberg–Marquardt iteration method is to solve Equation (16), where *J* is the Jacobian matrix of the objective function, μ is the dynamic update factor, *I* is the identity matrix and *g* is the gradient vector of the objective function. The dynamic update factor is determined by the size of the objective function. When the objective function is larger, it is proven that the current gradient is toward the optimal solution. At this time, the dynamic update factor should be appropriately reduced, and the step size should be increased to speed up the solution. Otherwise, the dynamic update factor should be increased to reduce the step size and prevent solution divergence. The iterative updating equation is shown in Equation (17), and *T* is the coordinate transformation matrix (i.e., the iterative parameter):

(16) $$\left(J^{T}J+\mu I\right)\cdot \Delta \vec{p}=-g$$

(17) T=T+ΔT

### 2.2. NDT Mapping Process and Effect Display

**Figure 2:**
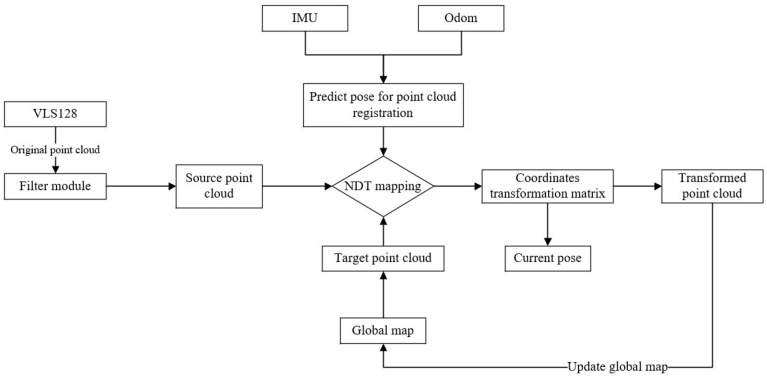
NDT mapping flow chart.

The NDT mapping method is a scan-to-map method [26], where scan refers to the current frame point cloud scanned by LiDAR while map refers to the target point cloud map formed by splicing historical frames frame by frame. Due to the limited scanning range of LiDAR, one scan cannot include all environmental information. Therefore, it is necessary to use LiDAR to continuously scan and collect environmental information, fix the target point cloud map and splice the collected laser point cloud to the target point cloud map frame by frame through the coordinate transformation matrix. The whole mapping process is shown in Figure 2.

In this paper, the Velodyne 128 line of LiDAR is used to collect the original point cloud information. After voxel filtering, the source point cloud is obtained and input into the NDT mapping module. We take the input point cloud of the first frame as the target point cloud map (i.e., the initial global map). We then fix the global map and input it into the NDT mapping module as the target point cloud. The initial coordinate transformation matrix is provided for the NDT mapping module through the IMU and Odom. The NDT mapping module performs Levenberg–Marquardt iteration based on the initial coordinate transformation matrix after the input of the source point cloud and the target point cloud, and it outputs the optimal coordinate transformation matrix. Through the optimal transformation matrix, the laser point cloud of the current frame can be spliced with the global map, the global map can be updated, and the position of the whole vehicle relative to the coordinate origin of the target point cloud map can be obtained. Through the above operations, the laser point cloud of the current frame is continuously spliced to the global map, and finally the global point cloud map is built.

**Figure 3:**
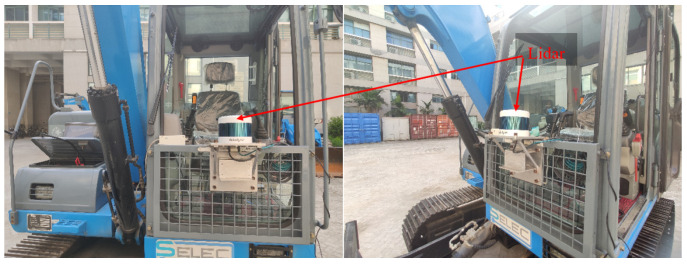
Point cloud collection equipment.

This paper selects an open field in the campus, uses the electric crawler excavator equipped with the Velodyne 128 line of LiDAR to collect environmental information and builds a map of the scene. The acquisition equipment is shown in Figure 3.

**Figure 4:**
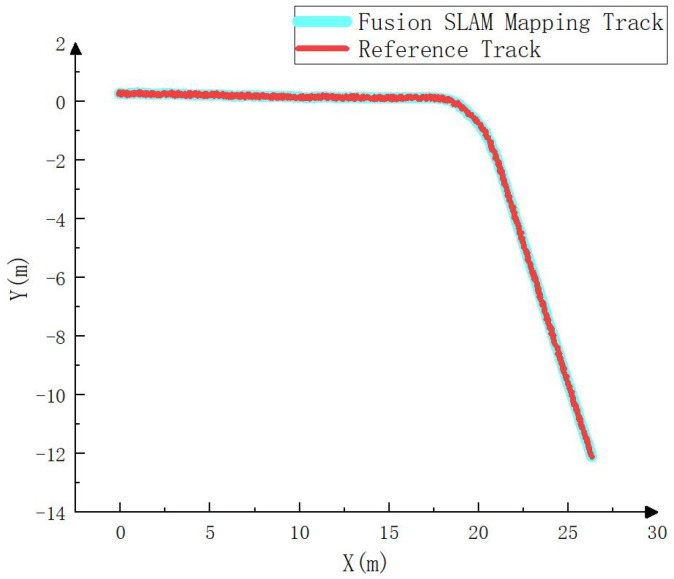
Comparison between the mapping track and the reference track.

**Figure 5:**
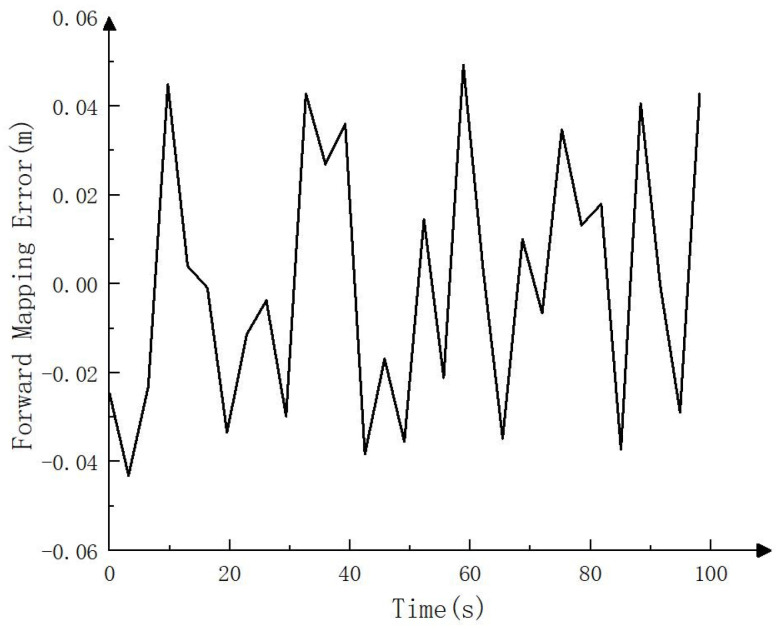
Forward mapping error (*x* axis direction).

**Figure 6:**
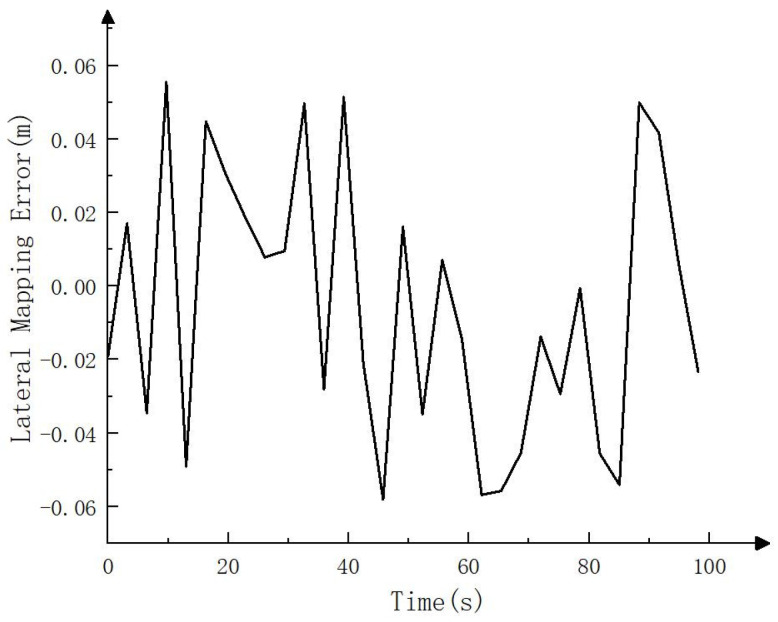
Lateral mapping error (*y* axis direction).

**Figure 7:**
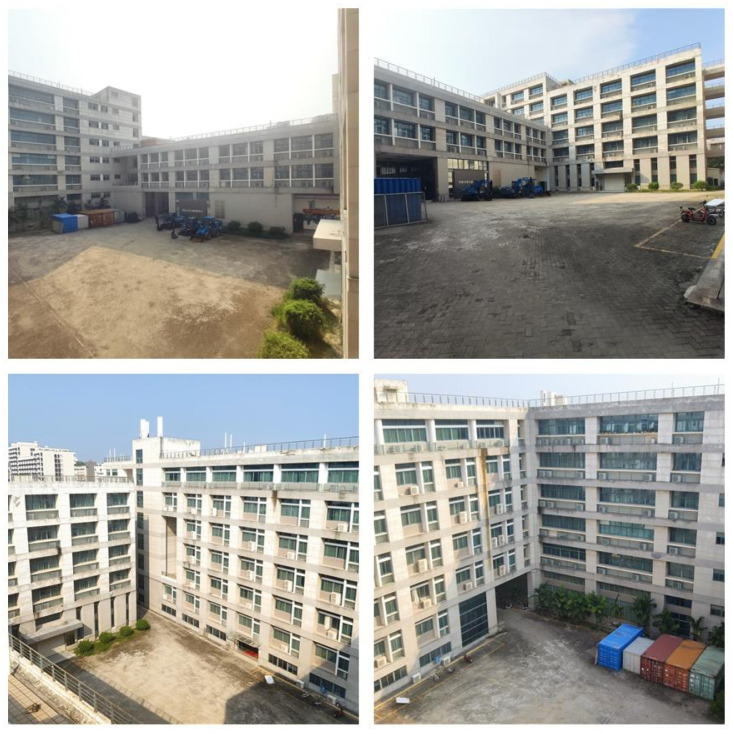
Mapping scene.

**Figure 8:**
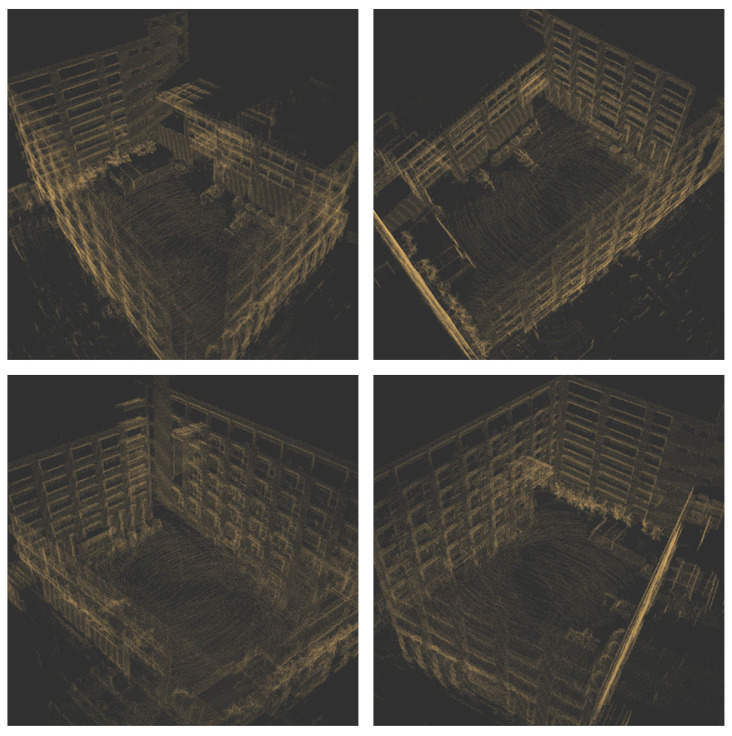
Point cloud map established for the mapping scene.

To compare the error between the mapping track and the actual track, this paper carries RTK on the electric crawler excavator, uses the RTK settlement result as the actual track (i.e., the reference track) and compares it with the mapping track settled by NDT. The comparison result is shown in Figure 4, Figure 5 and Figure 6. As can be seen in Figure 4, the mapping track of NDT settlement is basically consistent with the reference track. Figure 5 and Figure 6 show the forward (*x* axis direction) error and the lateral (*y* axis direction) error between the mapping track and the reference track, respectively. According to Figure 5 and Figure 6, the maximum error between the mapping track and the reference track in the forward direction was 0.05 m, the average error was 0.025 m, and the root mean squared error was 0.029 m. Meanwhile, the maximum error in the lateral direction was 0.06 m, the average error was 0.032 m, and the root mean squared error was 0.037 m. The experimental results show that the fusion SLAM method proposed in this paper can achieve high mapping accuracy in small- and medium-sized scenes. Figure 7 shows the mapping scene, and Figure 8 shows the point cloud map established for the environment through NDT.

### 2.3. NDT Positioning Process and Effect Display

**Figure 9:**
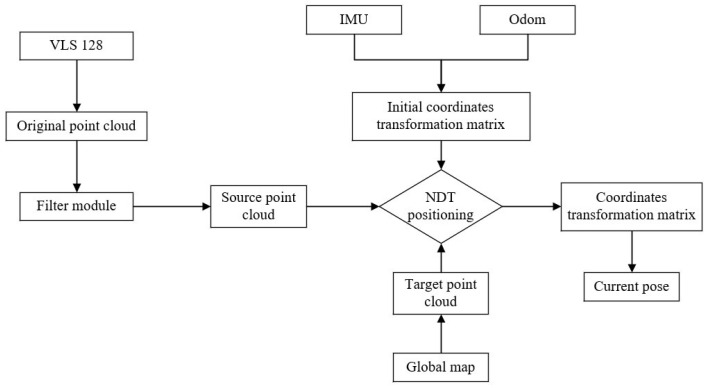
NDT positioning flow chart.

The NDT positioning process is similar to the NDT mapping process, except that there is no link to update the global map. The current frame point cloud scanned by LiDAR is sampled into the source point cloud through voxel filtering and matched with the global map. The initial coordinate transformation matrix is still provided by the IMU and Odom. The optimal coordinate transformation matrix is continuously optimized by the Levenberg–Marquardt iteration method so as to output the current position and attitude and realize the positioning of the whole vehicle. Figure 9 shows the overall flow chart of NDT positioning.

**Figure 10:**
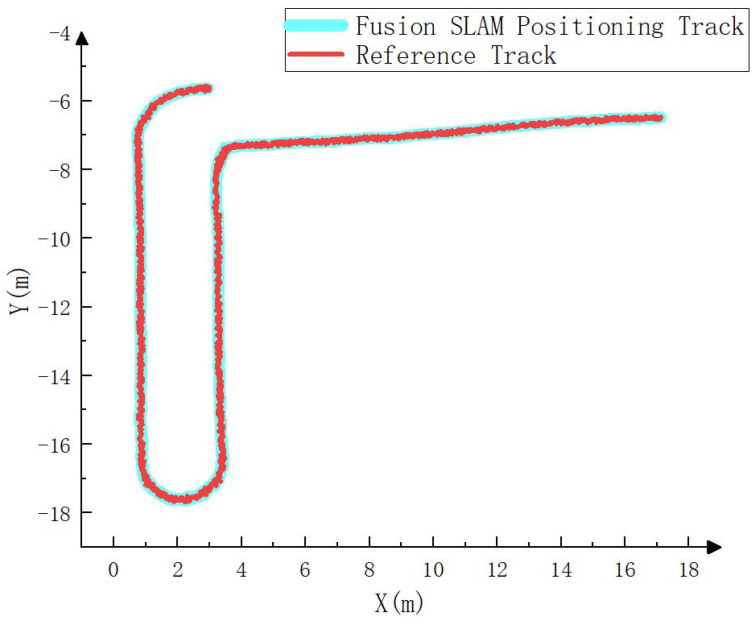
Comparison between the positioning track and the reference track.

**Figure 11:**
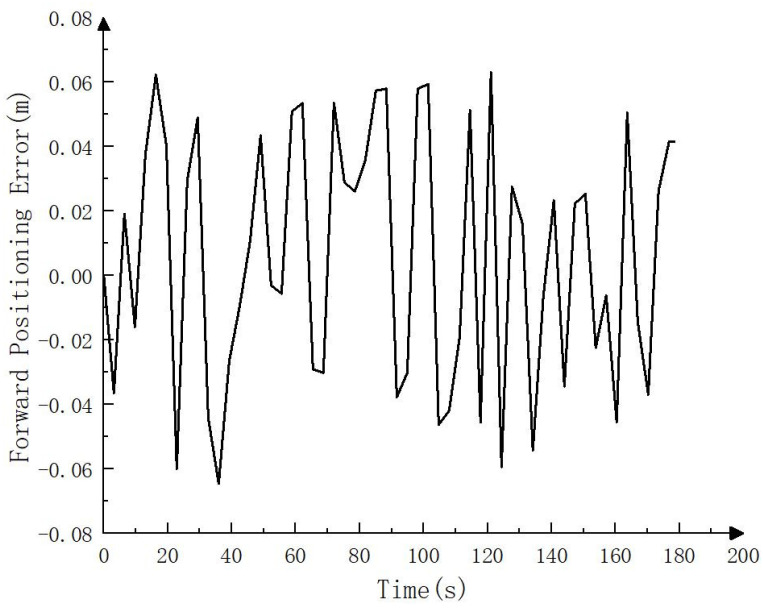
Forward positioning error (*x* axis direction).

**Figure 12:**
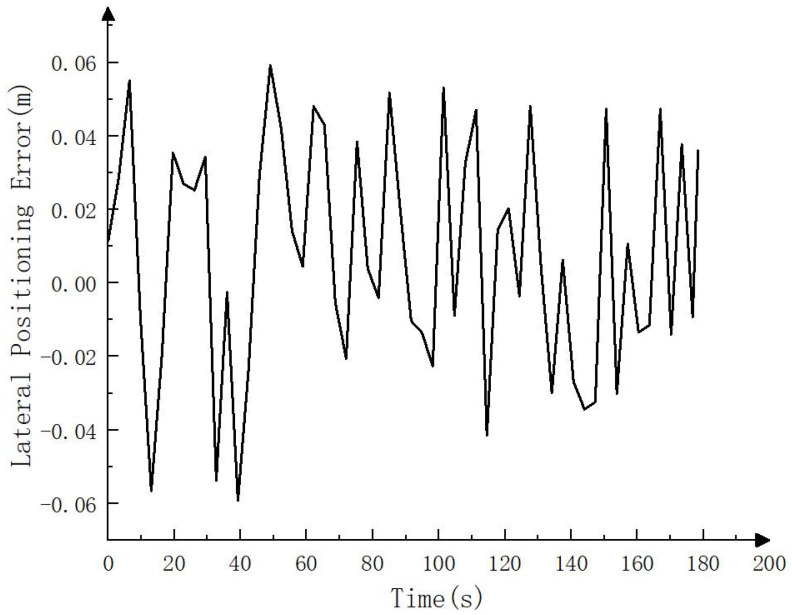
Lateral positioning error (*y* axis direction).

To compare the error between the fusion SLAM positioning track and the real track, the positioning track calculated by RTK is regarded as the real track (i.e., the reference track) and compared with the positioning track calculated by the fusion SLAM method. The results are shown in Figure 10, Figure 11 and Figure 12. As can be seen from Figure 10, the positioning track obtained by the fusion SLAM method was basically consistent with the reference track. Figure 11 and Figure 12 show the forward (*x* axis direction) error and lateral (*y* axis direction) error between the positioning track and the reference track, respectively. According to Figure 11 and Figure 12, the maximum error between the positioning track and the reference track in the forward direction was 0.065 m, the average error was 0.034 m, and the root mean squared error was 0.04 m. In addition, the maximum error in the lateral direction was 0.06 m, the average error was 0.032 m, and the root mean squared error was 0.032 m. The experimental results show that the fusion SLAM method proposed in this paper can basically meet the real-time positioning of driverless construction machinery in small- and medium-sized scenes and has high robustness.

## 3. Motion Control System of Crawler Construction Machinery Based on the Improved Pure Pursuit Algorithm

At present, the commonly used motion control algorithms in the field of unmanned driving include the pure pursuit algorithm [27], Stanley algorithm [28], linear quadratic regulator (LQR) algorithm [29] and model predictive control (MPC) algorithm [30]. Among them, the pure pursuit algorithm and Stanley algorithm are motion control algorithms based on vehicle kinematics which are applicable to low-speed vehicle motion control. The LQR and MPC algorithms are motion control algorithms based on vehicle dynamics which are suitable for high-speed vehicle motion control. Considering that tracked construction machinery belongs to low-speed mobile machinery, the dynamic model is relatively complex and difficult to establish, and the driving path has curvature discontinuity and cannot meet the conditions for using the Stanley algorithm. Therefore, the pure pursuit algorithm was adopted as the motion control algorithm of tracked construction machinery in this paper, which has the advantages of a simple principle, high robustness and no strict requirements for the driving path. The pure pursuit algorithm is mainly used for trajectory tracking of low-speed vehicles [31]. The existing pure pursuit algorithm was designed based on the Ackerman steering model and cannot meet the control requirements of crawler construction machinery. At present, there is no suitable unmanned motion control scheme for crawler construction machinery for reference. Therefore, this paper proposes an improved pure pursuit algorithm based on the motion characteristics of the crawler construction machinery so that it can be applied to the given trajectory tracking control of the crawler construction machinery.

**Figure 13:**
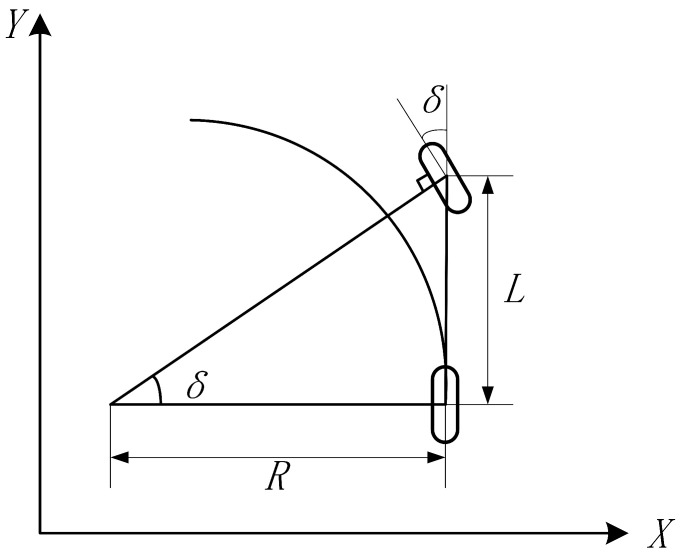
Kinematic model of bicycle.

The existing pure pursuit algorithm simplifies Ackerman steering to a bicycle model [32], as shown in Figure 13. With the center of the rear axle of the bicycle as the tangent point and the longitudinal body as the tangent line, the rear wheel can always move forward along the arc of the preview point by controlling the angle δ of the front wheel [33].

The relationship of the front wheel angle of bicycle can be obtained through a geometric relationship:

(18) $$\tan\left(\delta \right)=\frac{L}{R}$$

where *L* is the wheelbase of the front and rear wheels and *R* is the radius of the motion path.

**Figure 14:**
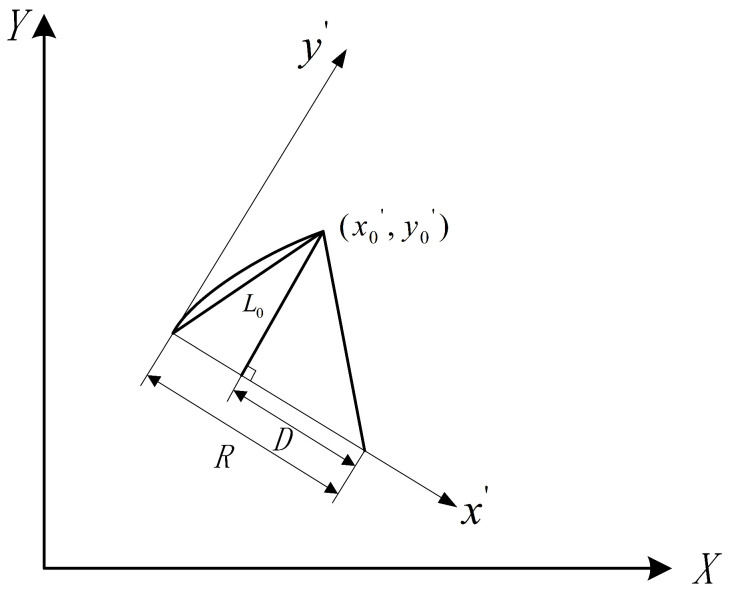
Analysis of pure pursuit geometric model.

According to Equation (18), since the wheelbase *L* of the front and rear wheels is known, when the radius R of the motion path is calculated, the corresponding rotation angle δ can also be calculated. Pure pursuit is a method based on a geometric relationship, as shown in Figure 14.

Here, $x^{{}^{\prime }}$ and $y^{{}^{\prime }}$ are the coordinate axes in the vehicle coordinate system, $\left(x_{0}^{{}^{\prime }},y_{0}^{{}^{\prime }}\right)$ represents the coordinates of the preview point in the vehicle coordinate system, $L_{0}$ is the distance from the origin of the vehicle coordinate system to the preview point, *R* is the radius of the motion path, and *K* is the curvature of the motion path. Based on the above geometric model relationship, the following derivation is made:

(19) $$D+x_{0}^{{}^{\prime }}=R$$

(20) $$D^{2}+y_{0}^{{}^{\prime }2}=R^{2}$$

(21) $$x_{0}^{{}^{\prime }2}+y_{0}^{{}^{\prime }2}={L_{0}}^{2}$$

(22) $$R=\frac{{L_{0}}^{2}}{2x_{0}^{{}^{\prime }}}$$

(23) $$K=\frac{1}{R}$$

**Figure 15:**
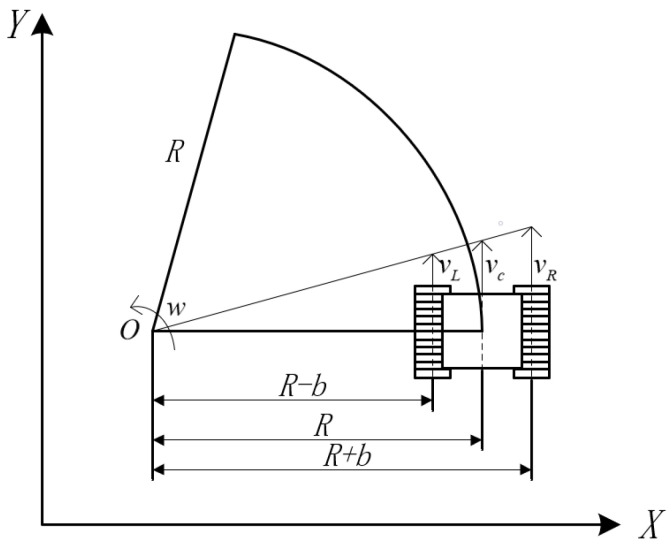
Kinematic model of the crawler construction machinery.

Based on the above analysis, the angle δ of the front wheel of the vehicle can be obtained by finding the radius *R* of the path between the current vehicle position and the preview point. Given the vehicle speed and planned waypoints, motion control of the vehicle based on Ackerman steering can be realized. The crawler construction machinery is different from ordinary Ackerman steering vehicles. Its motion control quantity is the travel speed of the left and right tracks. When the travel speed of the left track is greater than that of the right track, the crawler construction machinery turns right; otherwise, it turns left. When the left and right tracks travel at the same speed, the crawler construction machinery goes straight [34]. The kinematic model of crawler construction machinery is shown in Figure 15 [35]. This article takes the geometric center point of tracked construction machinery as the tangent point of the travel path, and by controlling the speed of the left and right tracks, the geometric center point of tracked construction machinery always travels along the planned path.

Here, *R* is the radius of the crawler construction machinery movement path, which can be solved by Equations (19)–(22), and *b* is the distance between the central axis of the left track (right track) and the central axis of the whole vehicle. Given the forward speed $v_{c}$ of the crawler construction machinery, the following derivation can be made based on the kinematic model of the crawler construction machinery [36]. Based on the above analysis, the following derivation is made. As the process of solving the arc radius *R* of the path is consistent with traditional pure tracking, the derivation will not be repeated here.

An excavator turning left is calculated as follows:

(24) $$w=\frac{v_{c}}{R}=\frac{2x_{0}^{{}^{\prime }}v_{c}}{L_{0}^{2}}$$

(25) $$v_{L}=w\left(R-b\right)=\frac{w(L_{0}^{2}-2x_{0}^{{}^{\prime }}b)}{2x_{0}^{{}^{\prime }}}$$

(26) $$v_{R}=w\left(R+b\right)=\frac{w(L_{0}^{2}+2x_{0}^{{}^{\prime }}b)}{2x_{0}^{{}^{\prime }}}$$

An excavator turning right is calculated as follows:

(27) $$w=\frac{v_{c}}{R}=\frac{2x_{0}^{{}^{\prime }}v_{c}}{L_{0}^{2}}$$

(28) $$v_{L}=w\left(R+b\right)=\frac{w(L_{0}^{2}+2x_{0}^{{}^{\prime }}b)}{2x_{0}^{{}^{\prime }}}$$

(29) $$v_{R}=w\left(R-b\right)=\frac{w(L_{0}^{2}-2x_{0}^{{}^{\prime }}b)}{2x_{0}^{{}^{\prime }}}$$

An excavator moving straight is calculated as follows:

(30) $$v_{L}=v_{R}$$

where *w* is the angular speed of the crawler construction machinery rotating around the center *O* of the motion path, $v_{L}$ is the running speed of the left track and $v_{R}$ is the running speed of the right track. Thus far, the formula derivation of the improved pure pursuit algorithm for crawler construction machinery has been completed.

## 4. Simulation Platform Construction and Real Vehicle Test

**Figure 16:**
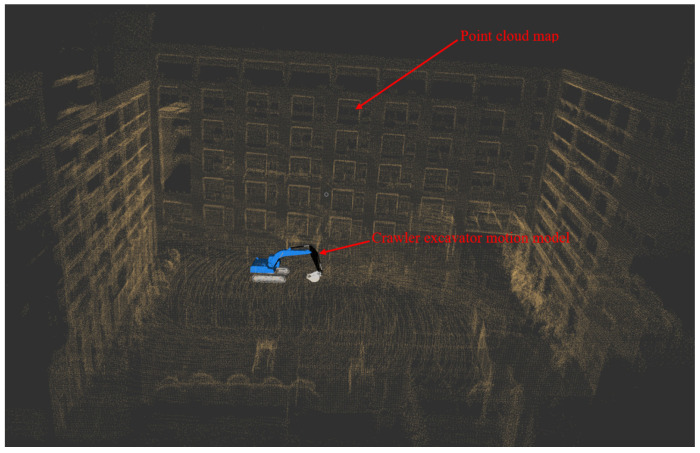
Simulation platform of electric crawler excavator.

**Figure 17:**
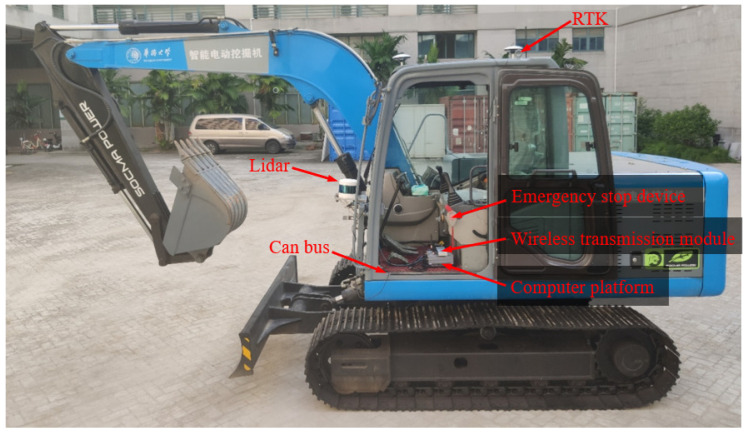
Driverless platform of electric crawler excavator.

**Figure 18:**
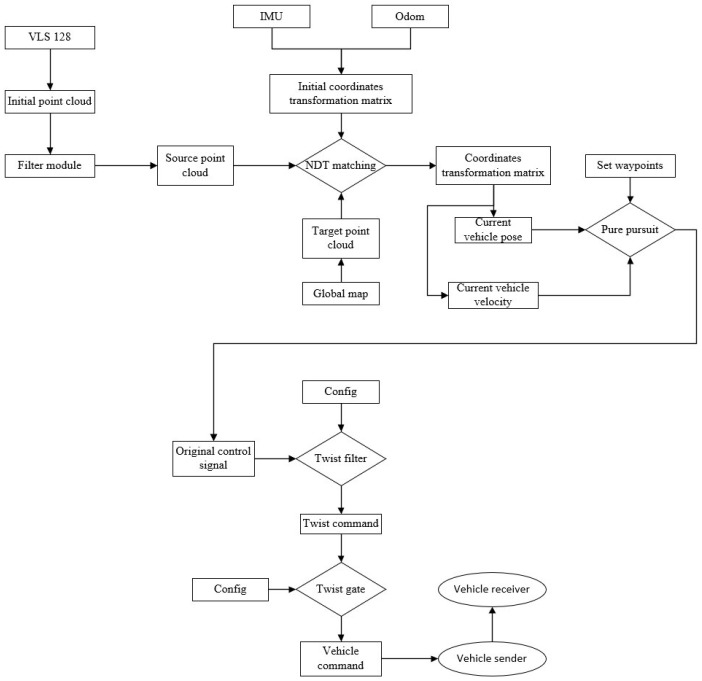
Flow chart of established trajectory tracking technology.

To verify the feasibility of the walking scheme based on the fusion SLAM and improved pure pursuit algorithms, the electric crawler excavator simulation platform and driverless platform were built as shown in Figure 16 and Figure 17. The verification idea of this paper was to build a target path through the waypoint file under the constructed map coordinate system, input the waypoint file and real-time positioning of the whole vehicle obtained through the fusion SLAM method into the improved pure pursuit algorithm and smooth the output control signal before sending it to the whole vehicle in the form of a CAN message. Finally, the tracking of the given trajectory was completed. The overall process is shown in Figure 18.

**Figure 19:**
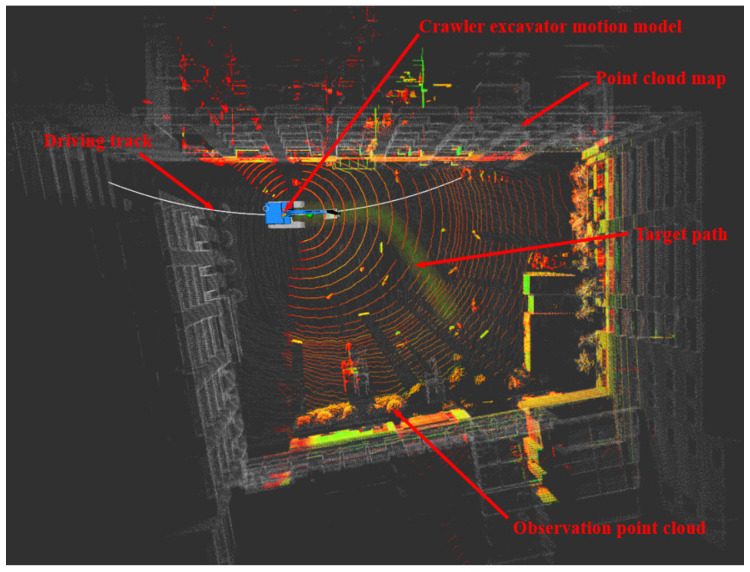
Visualization of simulation results.

Considering the safety of the test, this paper first verifies the walking scheme through the simulation platform. The simulation platform visualizes the simulation results with the help of the integration module rviz in ROS, and the whole vehicle motion model was built by using the Xacro macro language and SolidWorks. See Figure 19 for details.

The green path in Figure 19 is the target path, the white point cloud is the point cloud map constructed in advance, and the color point cloud is the historical observation point cloud collected through LiDAR. The blue crawler excavator is the motion model of crawler excavator established in this paper. The white path is the driving track (i.e., the path between the current position of the whole vehicle and the preview point). The driving track would constantly change with the change in the preview point so as to complete the tracking of the whole target path.

**Figure 20:**
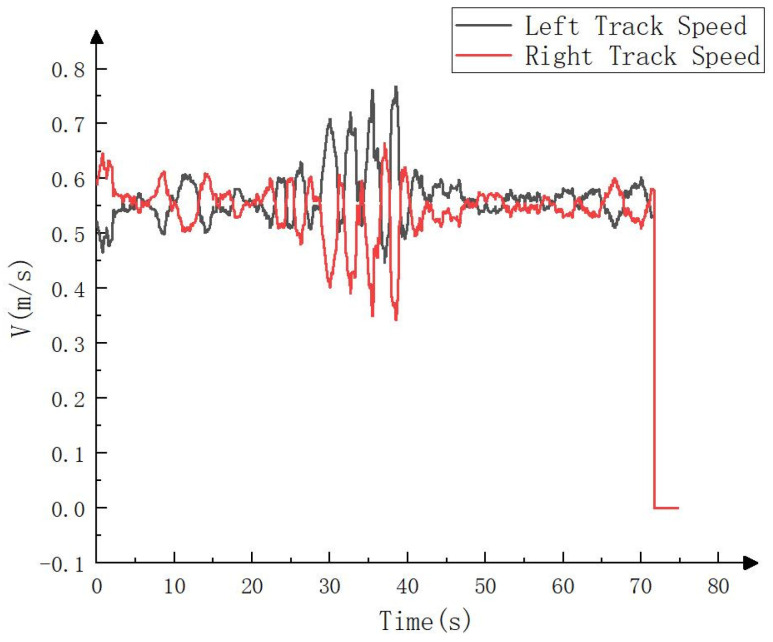
Left and right tracks’ speed in simulation process.

In this paper, constant speed tracking was adopted (i.e., the speed was constant in the tracking process). The vehicle speed set in the simulation process was 2 km/h (i.e., 0.56 m/s). The real-time positioning of the whole vehicle could be obtained by matching the observation point cloud with the point cloud map. The speeds of the left and right tracks could be obtained by inputting the position and attitude of the whole vehicle and the waypoint information of the target path into the improved pure pursuit algorithm and then smoothing it, as shown in Figure 20.

**Figure 21:**
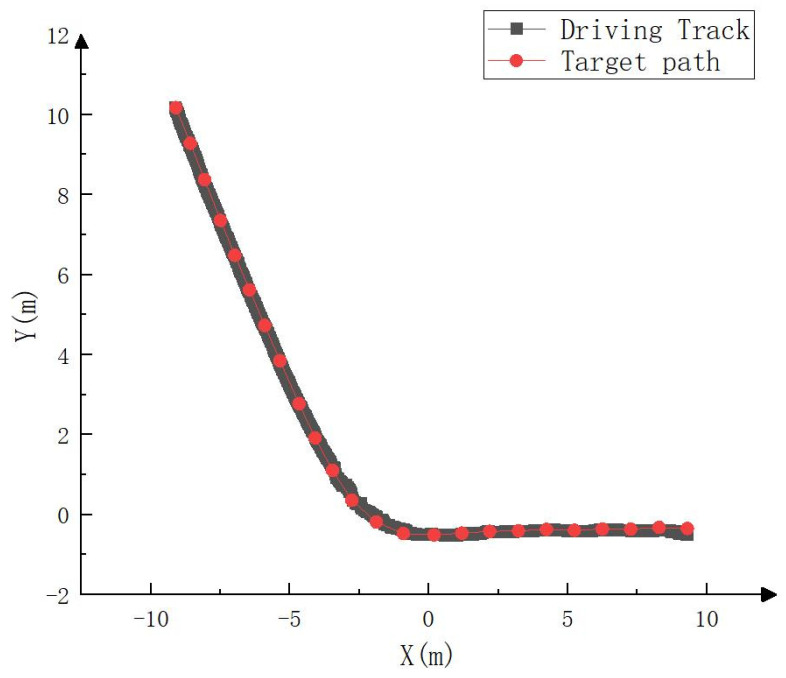
Comparison between simulated driving track and target path.

**Figure 22:**
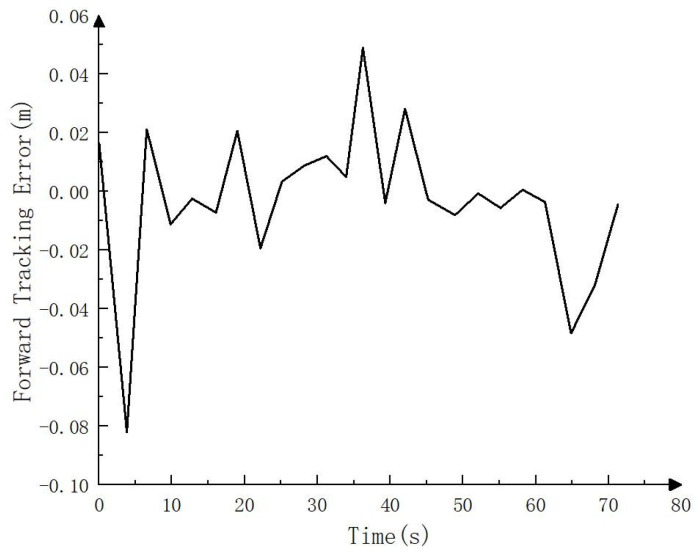
Forward tracking error (*x* axis direction) in simulation process.

**Figure 23:**
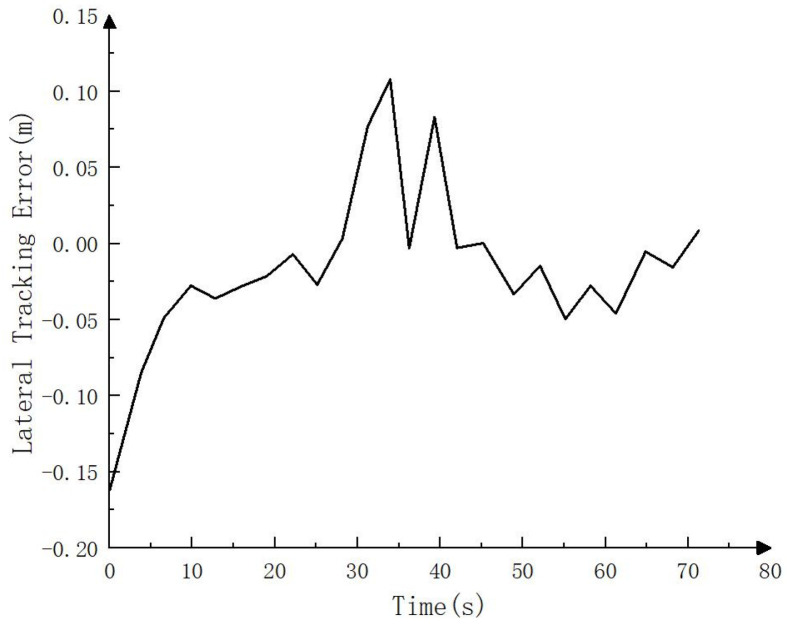
Lateral tracking error (*y* axis direction) in simulation process.

Since RTK could not be accessed in the simulation environment, to test the simulation tracking effect, the trajectory calculated by the fusion SLAM method was taken as the whole vehicle driving trajectory and compared with the target path, as shown in Figure 21, Figure 22 and Figure 23. As can be seen in Figure 21, the driving track was basically consistent with the target path in the simulation process. Figure 22 and Figure 23 show the forward (*x* axis direction) error and the lateral (*y* axis direction) error between the driving track and the target path, respectively. According to Figure 22 and Figure 23, the maximum error between the driving track and the target path in the forward direction was 0.082 m, the average error was 0.017 m, and the root mean squared error was 0.025 m. In addition, the maximum error in the lateral direction was 0.162 m, the average error was 0.038 m, and the root mean squared error was 0.055 m. The simulation results basically prove the feasibility of the driverless walking method for crawler construction machinery proposed in this paper.

**Figure 24:**
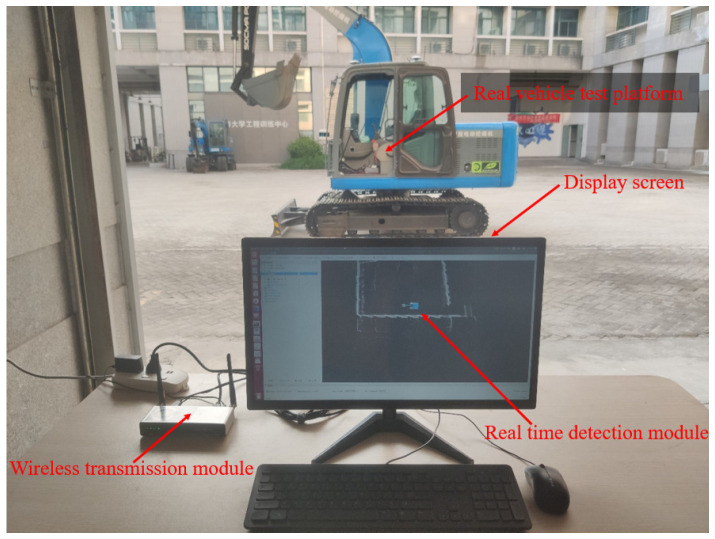
Real-time monitoring platform for real vehicle test.

**Figure 25:**
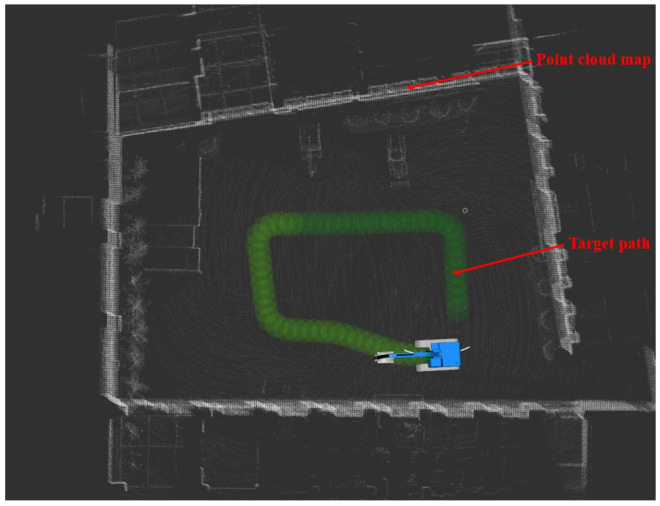
Real vehicle test target path planning.

**Figure 26:**
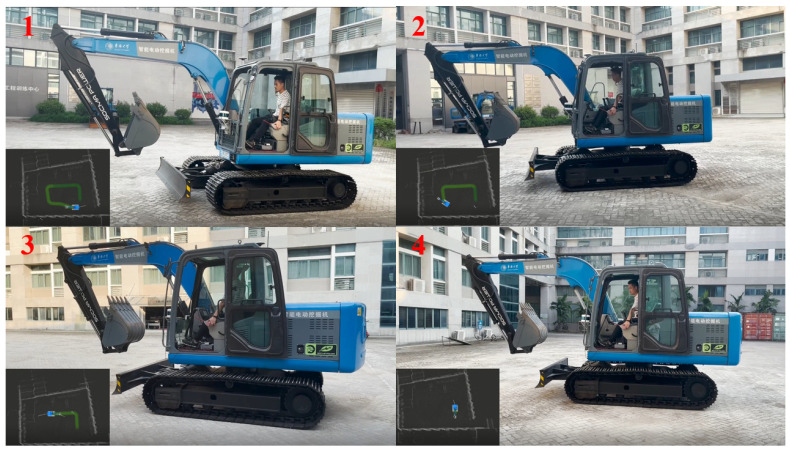
Overall process of real vehicle test.

To further verify the feasibility of the driverless walking method of crawler construction machinery proposed in this paper, a real vehicle tracking walking test was carried out based on the driverless platform of the pure electric crawler excavator built in this paper. Through the integration module rviz in ROS, the information collected by the sensors of the driverless platform and the real-time running state of the vehicle test were visualized, and the visualized information was transmitted from the computing unit in the real vehicle test platform to the display screen in the operation room through wireless image transmission equipment. On the premise of ensuring the safety of the testers, the whole test process was monitored. See Figure 24, Figure 25 and Figure 26 for more details. The main equipment parameters of the actual vehicle test platform are shown in Table 1. The onboard computing platform we used was TW-T609, developed by Tuwei Technology Company, with a computing power of up to 32 TOPS, which can effectively meet the real-time operation and verification of various unmanned driving algorithms in real vehicles.

The green path in Figure 25 is the path that needs to be tracked in the real vehicle test in this paper. The white point cloud is the point cloud map constructed in advance. Due to the limited computing power of the on-board computing unit, the observation point cloud collected by LiDAR during the real vehicle test was not displayed on the rviz.

**Figure 27:**
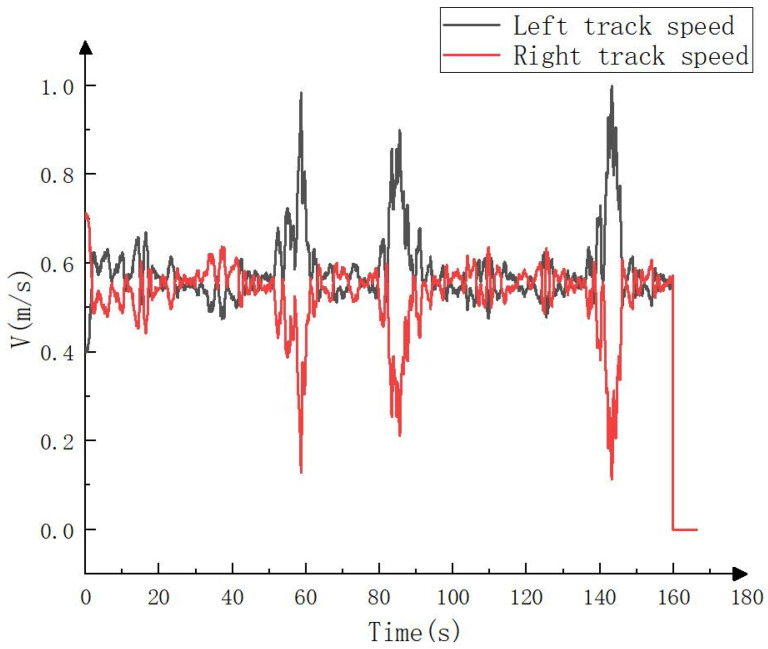
Left and right tracks’ speeds in real vehicle test.

The real vehicle test still adopted constant speed tracking, and the set vehicle speed was the same as that in the simulation test. The solution process of the left and right tracks’ speeds was also the same as that of the simulation test. The only difference was that the observation point cloud of the simulation test was recorded in advance by LiDAR, while the observation point cloud of the real vehicle test was obtained in real time with LiDAR. The calculateds speed of the left and right tracks are shown in Figure 27.

**Figure 28:**
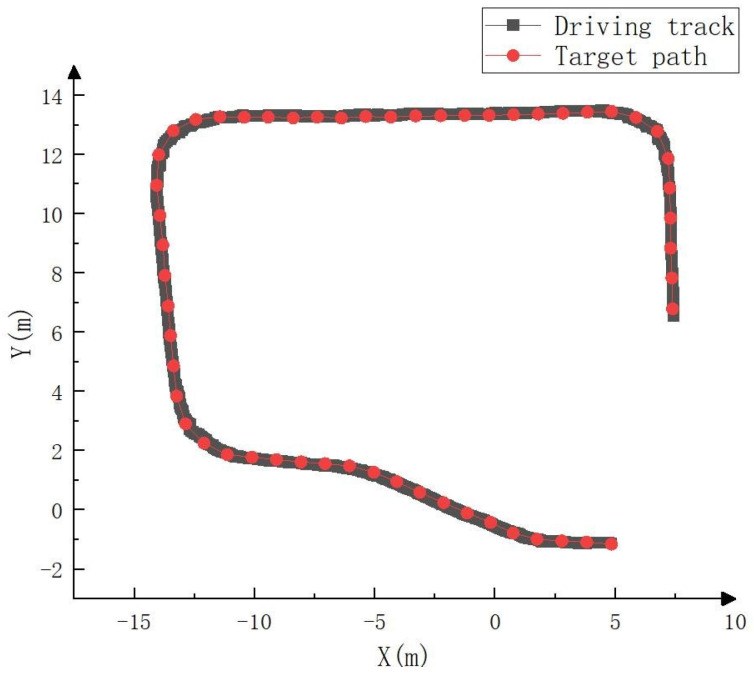
Comparison between driving track of real vehicle test and target path.

**Figure 29:**
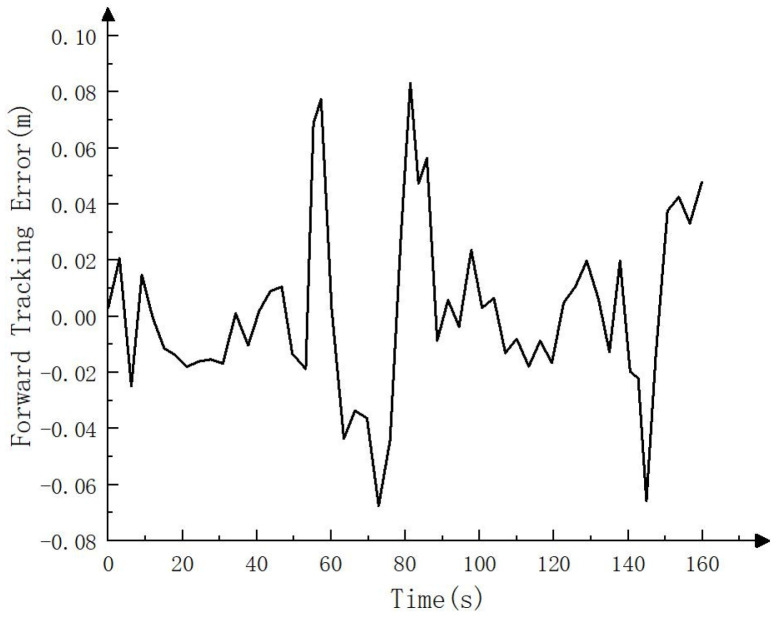
Forward tracking error (*x* axis direction) in real vehicle test.

**Figure 30:**
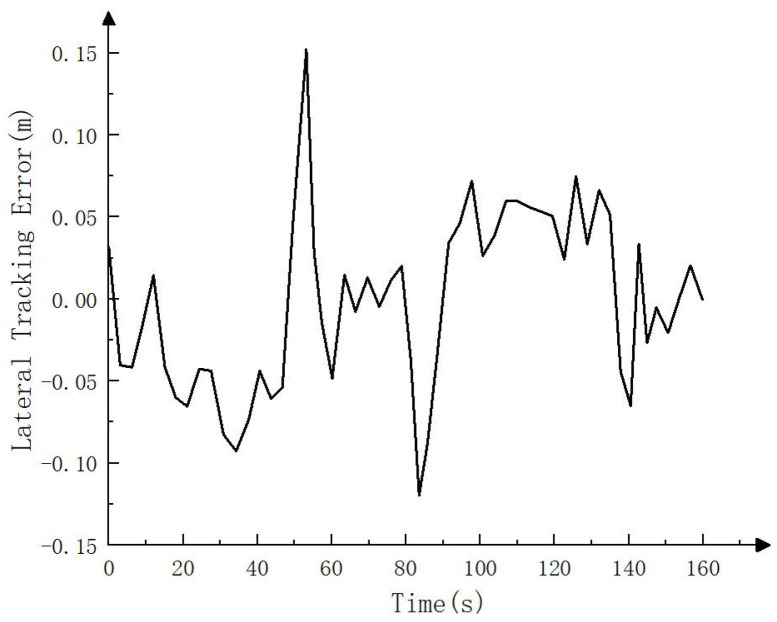
Lateral tracking error (*y* axis direction) in real vehicle test.

To test the actual tracking effect, RTK was connected to the whole vehicle. The trajectory calculated by RTK was regarded as the actual driving track and compared with the target path, as shown in Figure 28, Figure 29 and Figure 30. As can be seen in Figure 28, the driving track was basically consistent with the target path in the real vehicle test. Figure 29 and Figure 30 show the forward (*x* axis direction) error and lateral (*y* axis direction) error between the driving track of the real vehicle test and the target path, respectively. According to Figure 29 and Figure 30, the maximum error between the driving track of the real vehicle test and the target path in the forward direction was 0.083 m, the average error was 0.023 m, and the root mean squared error was 0.031 m. Additionally, the maximum error in the lateral direction was 0.152 m, the average error was 0.044 m, and the root mean squared error was 0.052 m. The real vehicle test results prove the feasibility of the crawler construction machinery driverless walking method based on the NDT fusion SLAM and improved pure pursuit algorithms again.

## 5. Conclusions

Aiming at the problem of weak GPS positioning signals in some working scenes of construction machinery, a fusion SLAM positioning system based on NDT was designed in this paper. Through multi-sensor fusion, the speed and quality of mapping were effectively improved. The test shows that the positioning system can still achieve more accurate vehicle positioning without GPS signal access. According to the walking characteristics of crawler construction machinery, this paper improved the existing pure pursuit algorithm to make it suitable for motion control of crawler construction machinery. Based on the NDT fusion SLAM and improved pure pursuit algorithms, this paper proposed a walking method suitable for driverless crawler construction machinery and built a crawler construction machinery driverless simulation platform and real vehicle test platform to carry out the corresponding simulation tests and real vehicle tests. The simulation and real vehicle test results show that the crawler construction machinery can track the set target path well through the walking method proposed in this paper. The driverless walking scheme of crawler construction machinery proposed in this paper fills the gap in the field of driverless construction machinery walking schemes to a certain extent and lays a certain foundation for the development of related technologies for driverless construction machinery.

Because the fusion SLAM method in this paper adopts the most basic NDT point cloud registration method, it can only achieve the accurate positioning of small-scale scenes. Later, we will consider improving the existing NDT point cloud registration method to meet the accurate positioning of large-scale scenes. The improved pure pursuit algorithm proposed in this article is currently only applicable to low-speed mobile construction machinery. In the future, we will consider the dynamics of construction machinery and develop new motion control algorithms to make it suitable for high-speed mobile construction machinery. At the same time, this paper only realized the trajectory tracking of the given path. Later, we will consider the introduction of perception and cost maps, combined with the hybrid A* algorithm, to realize the autonomous planning and tracking of construction machinery’s operation path. Hybrid A * [37] is a path planning algorithm that takes into account a vehicle’s kinematic constraints. Unlike the Dijkstra algorithm [38] and A * algorithm [39], it can generate a smooth path that conforms to a vehicle’s kinematic constraints, which helps to improve the smoothness of subsequent motion control. At the same time, we will use the PointPillars algorithm [40] and CenterPoint algorithm [41], commonly used in the industry as a perception scheme, and combine them with the common working conditions of construction machinery to create corresponding datasets for training in order to achieve a perception scheme suitable for construction machinery working conditions, providing more reference and basis for subsequent path planning, decision making and obstacle avoidance for construction machinery.

## Figures and Tables

**Table 1:** Main equipment parameters of the actual vehicle test platform.

Device Name	Equipment Model	Equipment Parameters
IMU	N100	Output frequency: 400 Hz
		Serial port baud rate: 921,600
		Pitch/roll accuracy: 0.05°
		number of axles: 9 axes
RTK/INS	CHCNAV CGI-610	Attitude accuracy: 0.1°
		Positioning accuracy: 1 cm
		Output frequency: 100 Hz
		Initialization time: 1 min
Lidar	Velodyne16	Measurement range: 300 m
		Measurement accuracy: ±3 cm
		Vertical measurement angle: 40°
		Horizontal measurement angle: 360°
		Measurement frequency: 5–20 Hz
On-board computer	TW-T609	CPU: 8-core Arm architecture
		GPU: 512 core Volte architecture + 64 Tensor core
		Computational power: 32 TOPS

## Data Availability

Not applicable.
